# High risk prescribing in older adults: prevalence, clinical and economic implications and potential for intervention at the population level

**DOI:** 10.1186/1471-2458-13-115

**Published:** 2013-02-07

**Authors:** Danijela Gnjidic, David G Le Couteur, Sallie-Anne Pearson, Andrew J McLachlan, Rosalie Viney, Sarah N Hilmer, Fiona M Blyth, Grace Joshy, Emily Banks

**Affiliations:** 1 Faculty of Pharmacy, University of Sydney, Bank Building A15, Science Rd, Sydney, NSW, Australia; 2 Centre for Education and Research on Ageing and Concord RG Hospital, Sydney, NSW, Australia; 3 Departments of Clinical Pharmacology and Aged Care, Royal North Shore Hospital and Kolling Institute of Medical Research, Sydney, NSW, Australia; 4 Sydney Medical School, University of Sydney, Sydney, NSW, Australia; 5 ANZAC Institute, Concord Hospital, Sydney, NSW, Australia; 6 Centre for Health Economics Research & Evaluation, University of Technology, Sydney, NSW, Australia; 7 National Centre for Epidemiology and Population Health, Australian National University, Canberra, ACT, Australia; 8 Sax Institute, Sydney, NSW, Australia

**Keywords:** High-risk prescribing, Prevalence, Clinical outcomes, Costs, Older adults

## Abstract

**Background:**

High risk prescribing can compromise independent wellbeing and quality of life in older adults. The aims of this project are to determine the prevalence, risk factors, clinical consequences, and costs of high risk prescribing, and to assess the impact of interventions on high risk prescribing in older people.

**Methods:**

The proposed project will utilise data from the 45 and Up Study, a large scale cohort of 267,153 men and women aged 45 and over recruited during 2006–2009 from the state of New South Wales, Australia linked to a range of administrative health datasets. High risk prescribing will be assessed using three indicators: polypharmacy (use of five or more medicines); Beers Criteria (an explicit measure of potentially inappropriate medication use); and Drug Burden Index (a pharmacologic dose-dependent measure of cumulative exposure to anticholinergic and sedative medicines). Individual risk factors from the 45 and Up Study questionnaire, and health system characteristics from health datasets that are associated with the likelihood of high risk prescribing will be identified. The main outcome measures will include hospitalisation (first admission to hospital, total days in hospital, cause-specific hospitalisation); admission to institutionalised care; all-cause mortality, and, where possible, cause-specific mortality. Economic costs to the health care system and implications of high risk prescribing will be also investigated. In addition, changes in high risk prescribing will be evaluated in relation to certain routine medicines-related interventions. The statistical analysis will be conducted using standard pharmaco-epidemiological methods including descriptive analysis, univariate and multivariate regression analysis, controlling for relevant confounding factors, using a number of different approaches.

**Discussion:**

The availability of large-scale data is useful to identify opportunities for improving prescribing, and health in older adults. The size of the 45 and Up Study, along with linkage to health databases provides an important opportunity to investigate the relationship between high risk prescribing and adverse outcomes in a real-world population of older adults.

## Background

In older adults, high risk prescribing can be defined as prescribing likely to lead to adverse clinical outcomes or prescribing that does not align with quality use of medicines principles. A range of indicators have been proposed to quantify high risk prescribing in older people. These include multiple medication use or polypharmacy, potentially inappropriate prescribing and exposure to high risk medicines, measured using different risk assessment tools.

Although the concomitant use of multiple medicines is often indicated in the treatment and prevention of health problems, polypharmacy is generally considered as high risk prescribing in older adults, and is associated with increased risk of harm [1-3], including adverse drug reactions, falls, hospitalisation, institutionalisation, and mortality [1]. Polypharmacy (concomitant use of ≥ 5 medicines) is common in older adults, and is estimated to be present in around half of Australians aged 65–74 years, and two-thirds of those aged ≥75 years [4]. The prevalence of “hyperpolypharmacy” (concomitant use of ≥ 10 medicines) in community-dwelling older adults has been reported to range from 5% to 26% [5,6].

High risk prescribing also includes potentially inappropriate prescribing, defined as the use of medicines whose potential harms to older adults may outweigh the benefits [7]. Potentially inappropriate prescribing can be assessed using explicit and implicit approaches. The Beers Criteria, an explicit USA consensus list is the most widely used indicator to define potentially inappropriate prescribing [7,8]. The associations between Beers Criteria and outcomes have been investigated across a range of populations and settings of older adults, with mixed findings being reported across studies [9]. Despite the efforts to improve the quality use of medicines in this population, recent systematic review suggests that potentially inappropriate prescribing is still common in older people [10].

High risk prescribing may also encompass exposure to “high-risk” medicines such as those with anticholinergic and sedative properties. A number of anticholinergic and/or sedative scores have been developed to capture the cumulative exposure to these pharmacological classes [2]. One such tool is the Drug Burden Index (DBI), a pharmacological score based on the principles of dose–response and cumulative effect, that measures an individual’s total exposure to medicines with anticholinergic and sedative effects [11]. In studies of older people, the prevalence of exposure to DBI medicines ranged from 29–70% [12,13]. Increasing DBI score has been associated with a higher risk of a range of adverse clinical outcomes, including functional impairment, frailty, falls and hospitalisation in populations of older adults from the USA, Australia and Europe [11-19].

Strategies to reduce high risk prescribing, including pharmacist-conducted medication reviews, educational interventions and policy approaches, have been trialled in older adults [20-22]. However, the success of these interventions has been limited. To reduce the likelihood of clinically significant adverse outcomes, rational withdrawal of medications may be the appropriate clinical decision and may result in significant clinical benefits in some older people with high risk prescribing [23]. To date, real-world approaches to reduce polypharmacy in older people have generally not been successful [22,24]. While interventional approaches can reduce medication exposure in older adults, the evidence for their clinical effectiveness and sustainability is conflicting [24].

The current evidence from pharmaco-epidemiological studies suggests that high risk prescribing increases the risk of adverse outcomes in older adults [1,2]. However, there is a lack of reliable evidence at the population level on the prevalence and outcomes relating to high risk prescribing in older people. Moreover, evidence is required regarding the impact of pragmatic medicines-related interventions aimed at improving medication management in older adults. The aims of this project are to determine the prevalence, risk factors, clinical implications and costs of high risk prescribing, and the impact of interventions on high risk prescribing in older people within a large population-based cohort study; specifically to:

1) Estimate the prevalence of high risk prescribing;

2) Investigate the risk factors for high risk prescribing;

3) Quantify the relationship of high risk prescribing to adverse health outcomes;

4) Determine whether associations between high risk prescribing and adverse outcomes vary according to individual and/or health care system specific factors;

5) Calculate the economic cost and implications of high risk prescribing to the health care system;

6) Investigate whether certain routine medicines-related interventions, including the Home Medicines Review and Chronic Disease Management are associated with reductions in high risk prescribing.

## Methods

### Overview of data sources

**Figure 1:**
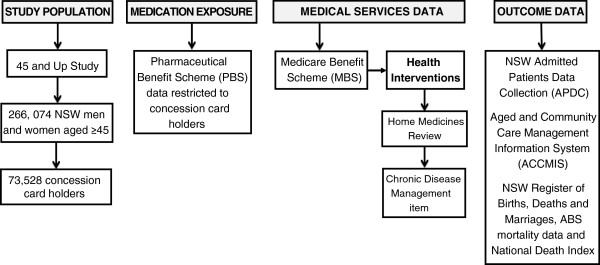
**Overview of the data sources. **Abbreviation: ABS, Australian Bureau of Statistics; NSW, New South Wales.

The proposed cohort analysis will utilise a population-based dataset combining self-reported questionnaire data (including data on life-style and social factors, functional capacity, physical activity, medical diagnosis and medication data) linked to routine administrative health datasets. This project will utilise the following data sources: the 45 and Up Study baseline questionnaire; the Australian Commonwealth Medicare Benefit Scheme (MBS) claims data, Pharmaceutical Benefit Scheme (PBS) claims data, Aged and Community Care Management Information System (ACCMIS) database, National Death Index; and New South Wales (NSW) datasets including the Admitted Patients Data Collection (APDC), and the Register of Births, Deaths and Marriages (Figure 1).

### The 45 and Up study cohort

The 45 and Up Study cohort includes 267,153 men and women aged 45 and over from the state of NSW, Australia, randomly sampled from the Medicare Australia database [25]. Participants completed a self-administered postal questionnaire and provided signed consent for participation and follow-up, including linkage to health records (including data on deaths, hospital admissions, and aged care admissions). Recruitment was conducted from February 2006 to April 2009, with an 18% response rate. Following exclusion of individuals with missing data on age and sex, 266,074 participants remained. Of these, 143,199 (54%) are aged 60 years and over (Table 1). Overall, 28% (n = 73,528) of the cohort reported holding a healthcare concession card, hereafter referred as concession card holders. This status will also be confirmed using the health administrative databases. For the purposes of the High Risk Prescribing project, the study sample will be restricted to cohort members who are concession card holders, as this allows full capture of health care utilisation.

**Table 1:** Characteristics of the concession card holders in the 45 and Up study, and total study population

**Characteristic**^**~**^	**Concession card holders (n = 73,528; 28%)**	**All participants (n = 266,074)**
***Socio-demographic factors***		
Age groups, n (%)		
45–59 years	14,888 (20%)	122,875 (46%)
60–69 years	25,599 (35%)	73,833 (28%)
70–79 years	20,432 (28%)	42,256 (16%)
≥ 80 years	12,609 (17%)	27,098 (10%)
Sex, n (%)		
Male	32,755 (45%)	123,402 (46%)
Female	40,773 (55%)	142,672 (54%)
Country of birth, n (%)		
Australia born	54,031 (73%)	199,342 (75%)
Born outside of Australia	19,497 (27%)	66,732 (25%)
Highest educational qualification, n (%)		
University degree	7,289 (10%)	61,290 (23%)
Certificate/diploma	21,932 (30%)	84,556 (32%)
Higher school/leaving certificate	6,757 (9%)	25,985 (10%)
School/intermediate certificate	20,338 (28%)	58, 611 (22%)
No certificate	15,358 (21%)	31,194 (12%)
Household income, n (%)		
Less than $20,000	31,380 (43)	52,359 (20%)
$20,000-$39,999	17, 260 (23%)	46,555 (17%)
$40,000-$69,999	5,545 (8%)	46,968 (18%)
$70,000 or more	1,007 (1%)	62,553 (24%)
Missing	18,336 (5%)	57,639 (22%)
Marital status, n (%)		
Married/defacto	46,771 (64%)	198,760 (75%)
Not married^*^	26,265 (36%)	65,703 (25%)
Region, n (%)		
Inner regional	29,909 (38%)	93,581 (35%)
Major cities	28,600 (39%)	119,792 (45%)
More remote	17,008 (23%)	52,640 (20%)
***Lifestyle and social factors***		
Alcohol use, number of drinks per week, n (%)		
None	31,163 (42%)	86,198 (32%)
1–14	31,400 (43%)	136,880 (51%)
≥ 15	8,517 (12%)	37,357 (14%)
Smoking history, n (%)		
Current smoker	6,833 (9%)	19,247 (7%)
Former smoker	28,365 (39%)	96,080 (36%)
Never-smoker	38,017 (52%)	149,900 (56%)
Body Mass Index, kg/m^2^, n (%)^#^		
Underweight (15–18.4 kg/m^2^),	1,139 (2%)	3,094 (1%)
Normal weight (18.5–24.9 kg/m^2^)	23,306 (32%)	90,563 (34%)
Overweight (25.0–29.9 kg/m^2^)	25,624 (35%)	97,163 (37%)
Obese (≥30.0 kg/m^2^)	16,761 (23%)	54,903 (21%)
Missing	6,698 (9%)	20, 351 (8%)
***Functional capacity and medical diseases***		
Needing assistance with daily tasks because of long-term illness or disability, n (%)		
Yes	8,113 (11%)	14,499 (5%)
No	60,529 (82%)	238,661 (90%)
Missing	4,886 (7%)	12,914 (5%)
Physical activity, tertile, n (%)		
1	22,087 (30%)	77,019 (29%)
2	23,422 (32%)	86,501 (33%)
3	21,986 (30%)	87,411 (33%)
Missing	6,033 (8%)	15,143 (6%)
Self-rated health, n (%)		
Excellent	5,705 (8%)	38,758 (15%)
Very good	20,449 (28%)	94,776 (36%)
Good	26,822 (36%)	86,711 (33%)
Fair	13,864 (19%)	30,822 (12%)
Poor	3,187 (4%)	5,625 (2%)
Missing	3,501 (5%)	9,328 (4%)
Kessler 10 score, psychological distress, n (%)		
Low	42,596 (58%)	180,524 (68%)
Moderate	10,223 (14%)	36,817 (14%)
High	4,623 (6%)	12,718 (5%)
Very high	2,427 (3%)	5,144 (2%)
Missing	13,659 (19%)	30,871 (12%)
Diabetes, n (%)		
Yes	10,716 (85%)	24,609 (9%)
No	62,812 (5%)	241,465 (91%)
Cardiovascular diseases, n (%)^^^		
Yes	17,157 (23%)	39, 815 (15%)
No	56,371 (77%)	226,259 (85%)
Cancer, n (%)^≠^		
Yes	11,258 (15%)	30,426 (11%)
No	62,270 (85%)	235,648 (89%)

Missing values given where they amount to 5% or more of the total.

^**~**^ Percentages do not add up to 100% in all instances due to missing values and rounding.

^*^Single, widowed or divorced.

^#^Body Mass Index categories in line with World Health Organization classifications.

^^^Heart disease, stroke or blood clot.

^≠^Self-reported history of cancer excluding skin cancer.

### Commonwealth administrative health datasets

#### MBS and PBS administrative databases

MBS and PBS are part of the Australia's national health insurance arrangements [26]. These schemes provide subsidised access to medical services and pharmaceuticals for Australian residents. The MBS database contains detailed information on Commonwealth subsidised claims for medical services performed outside the public hospital inpatient setting.

The PBS database contains information on Commonwealth subsidised claims for prescribed medicines listed on the Schedule of Pharmaceutical Benefits. There are two main groups of PBS beneficiaries, concession card holders and general beneficiaries. The PBS database records only PBS-listed prescription medicines that attract a government subsidy. Concession card holders are required to contribute a co-payment of $5.80 (as at January 1^st^ 2012) towards the cost of their medicines. Since all PBS medicines cost more than the concession card holder threshold, they will always attract a Commonwealth subsidy. Therefore medication data from concession card holders are captured consistently in the PBS database. However, the co-payment threshold for general beneficiaries is $35.40 (as at January 1^st^ 2012). There are a range of PBS-listed medicines that cost less than this co-payment threshold. When these medicines are dispensed to a general beneficiary, the general beneficiary is required to pay the full cost of the medicines and the government does not contribute to payment. Consequently, PBS-medicines falling below the co-payment were not captured in the PBS database for general beneficiaries. However, following a policy change, since April 2012 the PBS database now captures all PBS-dispensed medicines, regardless of whether the government contributes to the payment. To manage the potential incomplete capture of PBS-listed medicines dispensed to general beneficiaries we will restrict our analysis to healthcare concession card holders. Recent estimates indicate that over 90% of individuals aged 65 years and over hold a government health care concession card [27]. Therefore, the results will be highly generalisable to the general population of older Australians.

#### ACCMIS, National Death Index and Australian bureau statistics mortality data

The ACCMIS contains information on residential aged care services provided to older people who can no longer live at home [28]. The National Death Index dataset contains records of all deaths occurring in Australia since 1980 [29], along with data from the Australian Bureau of Statistics on cause of death.

### NSW health administrative datasets

#### APDC and NSW registry of births and death databases

The APDC is a complete census of all services for admitted patients provided by public hospitals, public psychiatric hospitals, public multi-purpose services, private hospitals and private day procedure centres in NSW, Australia [30]. Finally, the NSW Registry of Births and Death will be utilised to provide fact of death data.

### Linkage process

The data linkage procedures will be performed separately for Commonwealth and NSW databases as these health datasets are held by different jurisdictions. The linkage processes are summarised below.

#### Commonwealth datasets

As 45 and Up Study participants were sampled from the Medicare enrolment database and each was assigned an encrypted version of the unique Medicare ID, the linkage to PBS and MBS Commonwealth data is deterministic. PBS and MBS data for all 45 and Up Study participants have already been linked to baseline questionnaire data. The linkage with the National Death Index dataset, including the Australian Bureau of Statistics mortality data will be performed by the Australian Institute of Health and Welfare. Linkage of the ACCMIS database to the 45 and Up Study and other datasets through a third party using best practice privacy preserving protocols will be explored.

#### NSW datasets

The Centre for Health Record Linkage (CHeReL) routinely links identifying details (including full name, date of birth, sex and address) from 45 and Up Study participants to those from the NSW APDC and death records, to provide regular updates to events in the cohort. This is a part of an established program of linkage. Records are matched probabilistically using privacy-preserving current best practice with *ChoiceMaker* software [31], an algorithm based on maximum entropy theory to develop a predictive model that classifies record pairs.

### Medication exposure

Medication exposure will be measured using the 45 and Up Study predominantly using PBS dispensing data, validated and calibrated against self-reported medication use from the 45 and Up Study questionnaire. As the PBS database does not capture private prescriptions, medicines dispensed to public hospital inpatients, over-the-counter medicines and many complementary and alternative medicines, the extent to which PBS data reliably categorise high risk prescribing is not known. The authors are currently conducting the validation projects to estimate the extent and implications of any under-ascertainment of high risk prescribing using PBS data, compared with self-reported measures from the 45 and Up baseline questionnaire.

#### Assessment of high risk prescribing

High risk prescribing will be defined using three indicators: polypharmacy; Beers Criteria; and DBI. Polypharmacy will be defined as the concurrent dispensing of five or more medicines [32] over a six month period, and using the total number of medicines as a continuous variable. The updated 2010 Beers criteria will be used to assess potentially inappropriate prescribing [8]. The linked datasets will be screened for medicines and diagnosis considered by the updated Beers criteria. Data on six medical conditions included in the updated Beers criteria have been collected for the 45 and Up Cohort. Medicines will be used as a surrogate marker of other diseases considered by the updated Beers criteria [15]. Inappropriate prescribing will be considered present if the participant is prescribed at least one medicine included in the updated Beers criteria.

The DBI, a measure of exposure to drugs with anticholinergic or sedative effects will be calculated using the equation [11],

(1) $$\text{DBI}=\sum \left[D/\left(\delta +D\right)\right],$$

where *D* is the daily dose, and *δ* is the minimum efficacious dose (the minimum dose in the approved product information registered by the Australia’s Therapeutic Goods Administration, TGA), an estimate of the dose required to obtain 50% of the maximal effect. The daily dose will be estimated from the total quantity dispensed, using a standard pharmaco-epidemiological approach [33], by multiplying the strength (mg) and quantity dispensed, divided by the time period over which the medicines were taken during the study period. The total drug burden for the participant will be calculated as the sum of the drug burden of all anticholinergic or sedative medicines a participant is exposed to using a linear additive model. The Anatomical Therapeutic Codes (ATC) will be used to screen for anticholinergic and sedative drugs [34]. The minimum dose registered by the TGA will be obtained for all drugs with anticholinergic and sedative effects.

### Risk factors

Data on individual risk factors from the 45 and Up Study questionnaire (eg. age, gender, socioeconomic status, disability), and health system characteristics from linked data (eg. prescriber, number of pharmacies and geographical region) will be obtained to identify factors associated with the likelihood of high risk prescribing.

### Ascertainment of outcomes

Data on a range of clinical outcomes will be obtained, including: hospitalisation (first admission to hospital, total days in hospital, cause-specific hospitalisation, covering falls-related hospital admission, and fracture-related hospital admission); all-cause mortality; and, where possible, admission to institutionalised care and cause-specific mortality. Follow-up will be from the date of recruitment, to the last available data on hospitalisation, death or institutionalisation, whichever occurs first. The 45 and Up Study recruitment was from February 2006 to April 2009, giving a range of follow-up duration of 2–5 years, by end 2012 (accounting for linked data delays).

### Health interventions

Changes in high risk prescribing will be evaluated in relation to two health interventions: the Home Medicines Review [35], and the Chronic Disease Management [36] item of the MBS. The Home Medicines Review is a Commonwealth-funded service introduced in 2001. It is a free collaborative medication management service provided to the patient. The collaborative service involves the patient, their primary physician and pharmacist, whereby the pharmacist referred by the patient’s general practitioner conducts an interview with the patient and generates a written report for the primary physician to discuss the medication management plan with their patient. The Chronic Disease Management program was introduced in 2005 to enable primary care physicians to plan and coordinate the health care of patients with chronic or terminal medical conditions. A ‘chronic medical condition’ is one that has been or is likely to be present for at least six months, including but not limited to asthma, cancer, cardiovascular disease, diabetes mellitus, musculoskeletal conditions and stroke.

### Economic analysis

The cost analysis will be undertaken from a health system perspective. Total annual health system costs will be estimated overall and for each type of health care resource utilisation for each individual in the 45 and Up Cohort, based on their hospital, Medicare and pharmaceutical utilisation data. Costs will be assigned to each type of resource utilisation based on standard Australian sources (for example, using the PBS and MBS for pharmaceutical and medical services, national Australian Refined Diagnosis Related Groups cost weights for inpatient services and standard ambulatory costs for outpatient and emergency department visits). Differences in total and average annual costs will be estimated for the high-risk and non-high risk prescribing groups using each of the definitions of high risk prescribing. Covariates will be included in the cost equations and interacted with a dummy variable for the high risk prescribing group. Differences in total health care costs and for each type of health care resource utilisation will be estimated.

### Statistical analysis

The statistical analysis will be conducted using the standard pharmaco-epidemiological methods including descriptive analysis, univariate and multivariate regression analysis (eg. Cox regression modelling, negative binomial or Poisson regression), time-varying methods, and controlling for relevant confounding factors using a number of different approaches (eg. stratification by disease, propensity scores modelling).

To answer the first study objective, analysis will be performed to calculate the high risk prescribing indicators, using the 45 and Up Study cohort, linked to PBS data and restricted to concession card holders. The patterns of high risk prescribing will be summarised using proportions and means. To answer the second and third objectives, analysis will be performed to identify individual and health system based risk factors for high risk prescribing, using the same dataset in aim one. Univariate and multivariate analysis with relevant covariates will be used to quantify the risk for high risk prescribing compared to age and gender-matched controls. Using the same dataset, linked to other data sources, we will investigate the relationship of high risk prescribing indicators with hospitalisations, deaths and institutionalisation, allowing for a range of factors. Analysis related to the fourth objective – calculation of the economic costs and implications of high risk prescribing to the health care system – will be performed as outlined in the previous section. To answer the fifth objective, we will perform the analysis to evaluate the possible impact of policy-related interventions on high risk prescribing. In particular, we will compare the rates of high risk prescribing in participants with and without these interventions and the incidence of high risk prescribing before and after each of these interventions, within individuals.

### Sample size

Using data available to the end of 2012, the project will be highly powered for the main outcomes, with the ability to detect a minimum relative risk of death (the least common outcome) of 1.12, for each of the three high risk prescribing indices (assuming the following prevalences: DBI 48%; Beers 20% and polypharmacy 30%) [5,15], at 5% significance and 80% power. The incidence of total hospitalisation and hospitalisation for falls and fractures will be greater than the incidence of death, and hospitalisation for adverse drug reactions is similar to the incidence of death. To investigate the relationship between MBS interventions and outcomes, the linkage will be updated annually to increase power. An estimated 5% of people aged ≥ 65 have had a Home Medicines Review; however we are not able to locate estimates for other MBS review items. Assuming 5% exposure over the study period, for the smallest high risk prescribing stratum (Beers: 20%) this would allow detection of a relative change in prevalence of 0.05, comparing *before* and *after* the intervention.

### Ethical considerations

This study has been approved by the NSW Population and Health Services Research Ethics Committee (approval no. 00410). In addition, the 45 and Up Study also has overarching in principle ethical approval for data linkage from the University of New South Wales Human Research Ethics Committee (approval no. 05035), Sydney, Australia and the Department of Health and Ageing, Ethics Committee (approval no. 1/2005).

### Study cohort characteristics

The baseline characteristics of the 45 and Up study participants, and concession card holders are presented in Table 1. In this sample, 54% were female, 75% of participants were born in Australia and 75% were married or in a de facto relationship. In relation to lifestyle and social factors, 32% reported less than weekly alcohol use and 7% were current smokers. Of the total population, 37% were classified as overweight and 21% as obese. Excellent self-reported health was reported by the 15% of the population, and 68% had low psychological distress. In the 45 and Up Study cohort, concession card holders were generally older, had fewer educational qualifications, were more likely to be overseas born and not married, compared to other cohort members. In terms of functional capacity, concession card holders were more likely than non-card holders to report having a major disability, fair or poor self-reported health status, very high psychological distress and comorbidities.

## Discussion

High risk prescribing is a term used to describe a pattern of prescribing, which, for theoretical or empirical reasons, has been linked with adverse outcomes in older people. Identifying strategies to minimise high risk prescribing in older people requires a reliable evidence base on the magnitude of the problem and the relationship of high risk prescribing to adverse health outcomes, as well as the effectiveness of routine interventions to improve quality use of medicines. The Australian health system is well suited to this, because of a rich collection of large population-based and administrative databases. The size of the 45 and Up Study, along with linkage to routine health data will allow investigation of the relationship between high risk prescribing and adverse outcomes at the population level, including exploration of confounding, effect modification and intervention effects. In addition, opportunities to utilise other population-based datasets, including general PBS population and Department of Veteran Affairs’ data will also be explored.

It should be noted that the administrative datasets are not collected specifically to describe patterns of medicine utilisation or medical care and there are some important limitations to this cohort analysis, including generalisability, validity of data, bias and causality issues. Cohort studies often involve selected groups and produce results based on internal comparisons within the cohort. Theoretical and empirical work have shown these internal comparisons to be valid and reliable [37,38], including research from the 45 and Up Study [39]. The durability of findings from cohorts such as the British Doctors’ Study [40] is further testament to the robustness of this approach. The 45 and Up Study is not designed to be representative of the general population and the response rate to the baseline questionnaire was 18%, meaning that although relative risks calculated from internal comparisons, such as outcomes in relation to different categories of high risk prescribing, are valid and robust, caution should be exercised when generalising from the individual prevalences of exposure and other cohort attributes. Furthermore, variables from the 45 and Up Study questionnaire are self-reported and vary in their validity. However, the utilisation of established questionnaire instruments and population-level measures may minimise the possibility of bias [25].

While the gold standard for evaluating the causal effects of medicines on clinical outcomes is the randomised controlled trial, randomising older individuals to high risk prescribing would be inappropriate. Therefore, observational studies are essential to elucidate the association between high risk prescribing and health outcomes in this population, and to ensure the necessary degree of heterogeneity. However, the quantification of causality requires judicious interpretation of observational data [2]. A central issue is confounding, particularly by indication, in that individuals with high risk prescribing are likely to differ from others in ways that will affect disease risk. Awareness of this issue, along with using multiple analytic approaches to quantify potential biases is essential, particularly to examine the robustness of the findings with different assumptions and methods. Finally, it is important to avoid the assumption that any observed relationship of high risk prescribing to adverse outcomes is necessarily causal; a key focus of the project is to quantify variation in outcomes according to different patterns of medicines use, from the point of view of disease burden and consequences for the health system, rather than from a direct aetiological perspective.

In summary, the 45 and Up Study and its linkage with health databases will allow investigation of the relationship between high risk prescribing and adverse outcomes at the population level, which has not been possible in the past. The proposed study will generate an important, unique dataset for the quality use of medicines and will allow the identification of targets for improving prescribing in older people.

## Abbreviations

ACCMIS: Aged and Community Care Management Information System; APDC: Admitted Patients Data Collection; ATC: Anatomical Therapeutic Codes; CHeReL: Centre for Health Record Linkage; DBI: Drug Burden Index; MBS: Medicare Benefit Scheme; NSW: New South Wales; PBS: Pharmaceutical Benefit Scheme; TGA: Therapeutic Goods Administration.

## Competing interests

The authors declare that they have no competing interests.

## Authors’ contributions

DG and EB drafted the manuscript. EB, DLeC, SP, AM, RV, SH, DG, FB, and GJ contributed to the design of the study, and DLeC, SP, AM, RV, SH, FB, GJ were involved in reviewing the manuscript. All authors read and approved the final manuscript.

## Authors’ information

Danijela Gnjidic: Faculty of Pharmacy, Bank Building A15, Science Rd, University of Sydney, Camperdown, NSW 2006 Australia.

David Le Couteur: Centre for Education and Research on Ageing (CERA), Concord Hospital and University of Sydney, NSW 2139 Australia.

Sallie-Anne Pearson: Faculty of Pharmacy, Bank Building A15, Science Rd, University of Sydney, Camperdown, NSW 2006 Australia.

Andrew McLachlan: Faculty of Pharmacy, Bank Building A15, Science Rd, University of Sydney Camperdown, NSW 2006 Australia.

Rosalie Viney: Centre for Health Economics Research & Evaluation, PO Box 123, Broadway NSW 2007 Australia.

Sarah Hilmer: Royal North Shore Hospital and University of Sydney, Level 1, Acute Services Building, RNSH, St Leonards, NSW 2065 Australia.

Fiona Blyth: Centre for Education and Research on Ageing (CERA), Concord Hospital and University of Sydney, NSW 2139 Australia.

Grace Joshy: National Centre for Epidemiology and Population Health, Australian National University Canberra ACT 0200 Australia.

Emily Banks: National Centre for Epidemiology and Population Health, Australian National University Canberra ACT 0200 Australia.

## Pre-publication history

The pre-publication history for this paper can be accessed here:

http://www.biomedcentral.com/1471-2458/13/115/prepub
